# An ethical analysis of clinical triage protocols and decision-making frameworks: what do the principles of justice, freedom, and a disability rights approach demand of us?

**DOI:** 10.1186/s12910-022-00749-0

**Published:** 2022-02-11

**Authors:** Jane Zhu, Connor T. A. Brenna, Liam G. McCoy, Chloë G. K. Atkins, Sunit Das

**Affiliations:** 1 Temerty Faculty of Medicine, University of Toronto, Toronto, ON Canada; 2 Department of Anesthesiology and Pain Medicine, University of Toronto, Toronto, ON Canada; 3 Department of Political Science, Centre for Global Disability Studies, Scarborough College, University of Toronto, Toronto, ON Canada; 4 Centre for Ethics, University of Toronto, Toronto, ON Canada; 5 Division of Neurosurgery, St. Michael’s Hospital, 30 Bond Street, Toronto, ON M5B 1W8 Canada

**Keywords:** Resource allocation, COVID-19, Clinical triage protocols, Disability rights, Justice, Positive freedoms

## Abstract

**Background:**

The expectation of pandemic-induced severe resource shortages has prompted authorities to draft and update frameworks to guide clinical decision-making and patient triage. While these documents differ in scope, they share a utilitarian focus on the maximization of benefit. This utilitarian view necessarily marginalizes certain groups, in particular individuals with increased medical needs.

**Main body:**

Here, we posit that engagement with the disability critique demands that we broaden our understandings of justice and fairness in clinical decision-making and patient triage. We propose the capabilities theory, which recognizes that justice requires a range of positive capabilities/freedoms conducive to the achievement of meaningful life goals, as a means to do so. Informed by a disability rights critique of the clinical response to the pandemic, we offer direction for the construction of future clinical triage protocols which will avoid ableist biases by incorporating a broader apprehension of what it means to be human.

**Conclusion:**

The clinical pandemic response, codified across triage protocols, should embrace a form of justice which incorporates a vision of pluralistic human capabilities and a valuing of positive freedoms.

**Supplementary Information:**

The online version contains supplementary material available at 10.1186/s12910-022-00749-0.

## Background

Global emergencies require health care systems to shift into alternative operational states, modified to meet novel demands and overcome new constraints on resources. “Clinical Triage Protocols” are documents which serve to guide administrators and clinicians making decisions to partition and allocate care in such times of scarcity. The current global pandemic has been one such moment of resource strain requiring the development of these protocols.

The decisions made during triage are of great consequence. Current approaches to design clinical decision-making protocols prompted by the COVID-19/SARS-CoV-2 pandemic have been criticized as being discriminatory towards individuals of advanced age, or those with complex needs or medical comorbidities. These protocols highlight and potentially compound the disproportionate impact of acute health-system stressors on the lives of individuals who are already marginalized. For instance, likelihood of survival is used to support withholding care from those with a greater than 80% predicted mortality, which aggregates mortality from severe illness with confounders such as baseline cognitive impairment, neurodegenerative disease, or functional capacity [1, 2]. Clinical triage protocols highlight the systemic deprioritization of individuals with disabilities at the point of triage. Informed by a disability rights lens, we seek to (1) critically examine clinical triage frameworks and elucidate how they may highlight existing inequities; (2) propose ways in which these frameworks could be modified to fulfill obligations of justice; and (3) translate these principles beyond the COVID-19 context into decision-making within the arenas of medicine and public health more broadly. We propose that the disability critique of clinical guidance documents for triage directs us towards a richer conceptualization of justice beyond the utilitarian maximization of welfare, and towards justice as positive freedoms.

### The inequities of clinical triage frameworks

Many efforts to draft protocols that codify triage decision-making and guide practice during the current pandemic have faced scrutiny for intrinsically discriminatory policies [3–6]. In the draft Ontario COVID-19 Triage Protocol (2020), for instance, likelihood of survival is used to support the categorical exclusion of care for those with greater than 80% predicted mortality, including individuals with severe baseline cognitive impairment, inability to perform activities of daily living, advanced irreversible neurodegenerative disease, and decreased functional capacity as calculated by Clinical Frailty Scores [1, 2]. An open letter responding to this protocol highlighted its implicit deprioritization of individuals with cognitive or neurodegenerative disabilities [3]. Similar documents have evoked criticism in other jurisdictions (see Additional file 1: Table S1).

Although these clinical guidance efforts differ in scope, they share foci of *utility*, *maximization of benefits*, and *prioritization of clinical criteria* in the distribution of health care resources [1, 2, 7–9]. Despite the common guiding principle of utility, understood broadly as the obligation to produce a maximal balance of positive value over disvalue, there are gaps in how utility is operationally defined in these protocols. Unresolved questions include whether all life-years are to be valued equally, or whether there should be adjustment based on the age or quality of life of the patient. Similarly, it is unclear whether utility and quality of life are defined by a consistent set of criteria or relative to a patient’s pre-illness status. The guidance documents strongly advocate for the use of evidence-based and objective clinical criteria to direct triage and decision-making, and emphasize the need for clinical judgement to supplement triage protocols [9]. Several disability rights groups have objected to the disproportionate burden placed by these triage guidelines on individuals with disabilities, questioning the guidelines’ compatibility with human rights standards. Early in the pandemic, many disability rights groups rejected quality of life judgements, categorical exclusions, and long-term survival as metrics for allocating life-saving resources based on grounds of unjust discrimination [10]. However, a more controversial discussion is whether resources ought to be rationed and certain individuals ought to be deprioritized on the basis of *short-term survival* criteria. These criteria often involve the use of prediction scales such as the Clinical Frailty Score (CFS) or Sequential Organ Failure Assessment (SOFA).

The CFS, which is increased at baseline in individuals with difficulties in mobilizing or who require assistance to perform activities of daily living (ADLs), has been explicitly critiqued when applied to persons with disabilities. In a recent article, Atkins and Das argue that the CFS is overly reliant on “physical mobility as [an indicator] of physiologic reserve”, and does not account for chronic disease fluctuations, wrongly correlating care hours with health status. An assessment of medical frailty can thus conflate physical and social attributes with poor prognosis. [11] The association between increased predicted mortality with a need for assistance with ADLs and decreased functional capacity highlights one of the key points of the disability critique: that implicit assumptions exist about the categorical health of people with disabilities.

The SOFA score is another clinical instrument used to assign short-term mortality risk, comprising points assigned to various organ systems based on standardized metrics such as mean arterial pressure, required fraction of inspired oxygen, Glasgow Coma Scale, and serum measurements of creatinine, platelets, and bilirubin [12]. In addition to the relative inaccuracy of these scores in predicting mortality during COVID-19 [13], there has also been concern regarding their application for individuals with disabilities. For instance, the Glasgow Coma Scale (intended to measure severity of acute brain injury) may fail to account for the baseline status of individuals with impaired speech and motor abilities, including those with cerebral palsy and intellectual disability [10]. Features associated with stable underlying disabilities which do not predict short-term mortality may nonetheless correspond to higher scores on these scales, translating to a deprioritization of certain individuals—namely those living with disabilities.

Despite statutory, constitutional proclamations such as the United Nations Convention on the Rights of Persons with Disabilities, which forcefully aver the equality of persons with disabilities and their fundamental right to equal respect and consideration, the liberal perpetuation of ableist norms continues. Triage documents during the pandemic overtly valorize physical independence and autonomy, thus implicitly devaluing ‘dependent’ bodies. The eminent disability scholar Rosemarie Garland Thomson writes, in 2017: “… [T]he sparse literature on disability cultural competence mostly embraces the deficit model that predominates in bioethics and health care, thus reflecting a thin understanding of disability culture and disability equity.” [14].

Several disability rights activists, including Ari Ne’eman, insist that individualized assessment of patients, rather than appraisal of categorical impairment, constitutes a more ethically valid route for triage [10]. They conclude that disability activists have successfully influenced discussions about pandemic triage protocols even if revisions have not yet appeared. Nevertheless, we maintain that modern medicine continues to function within ableist social cultures. Moreover, it inherently seeks to prevent dysfunction and return people to a ‘normalized’ body. This effort often includes a presumption that a differently-bodied life is likely less capable, or enjoyable. Becoming more conscious of this internal bias can only assist bioethics in reducing the marginalization of disabled people.

## Main text

### Justice and triage

Justice, the ethical obligation to undertake fair arbitration of disputed claims, is one of the time-honoured, central principles of medical ethics [19]. Justice becomes particularly salient during widespread emergencies when it is threatened by an inability to provide beneficent care to everyone in need of it [16, 17]. *Distributive justice* [18] focuses on the fair distribution of limited resources: [19] in triage settings, it is employed to govern practice and existing triage guidelines [16, 20]. Justice is consequently invoked as a guiding principle in settings of resource allocation and population-level ethics, as highlighted by the call to *fairness* or *equity* in several clinical triage documents [3, 4].

The many criticisms laid against modern triage protocols warrant revisiting how we define justice in the frameworks that guide decision-making during resource scarcity. Distributive justice might advocate for *fair* allocation in the sense that everyone has equal access to resources, perhaps modified by severity of need, as has been suggested with respect to non-COVID treatment in the context of the COVID-19 pandemic [21]. However, it is often unclear in these contexts what principles such as *utility*, *proportionality*, *equity*, and *fairness* really mean, and whether these efforts incorporate facets of justice beyond utilitarianism.

## Exploring justice in the context of triage

We propose that reimagining the definition of justice in clinical triage protocols begins at the roots of these efforts, with the fundamental ethical and moral values that underpin them. Our goal is to address certain current decision-making frameworks which fail to take considerations of justice and equity seriously.

In the context of the COVID-19 pandemic, there have been several emerging suggestions about how we can best allocate limited resources [22]. It is critical, however, that these principles incorporate a defensible conception of justice which serves marginalized patients. Justice in triage is deceivingly complicated, and a model that privileges overall utility while systematically and disproportionately harming certain people is contestable. The disability critique, which reveals the shortcomings of traditional utilitarian undercurrents in clinical decision-making, invites a discussion of conceptualizations of justice which propose a richer notion of rights and freedoms, including but not limited to egalitarianism, fair equality of opportunity, and capabilities theory. In addition, the importance of procedural justice cannot be underestimated in the creation of triage guidelines.

### Prioritarian and egalitarian justice

The prioritarian model recognizes principles beyond utility (such as proportionality or reciprocity) and traditionally “non-clinical” features of patients including personal and social circumstance, as contributing to clinical state, giving “greater weight to those who are worse off” [23]. This approach is founded on the underlying assumption that stratifying individuals according to properties that are a function of a natural or social lottery (e.g., ability, ethnicity, gender, family upbringing) is unjust discrimination. The prioritarian model highlights the implicit bias in triage protocols criticized for explicitly excluding individuals with advanced neurocognitive disorders and unfairly assessing long-term survival of individuals with disabilities [3, 4]. Beyond defending against discrimination, egalitarian justice also argues for *positive* benefits for the disadvantaged. The *fair equality of opportunity* principle stipulates that individuals deprived of certain abilities should receive benefits that would “ameliorate the unfortunate effects of life’s lottery” [15]. In other words, fair equality of opportunity permits inequalities in the distribution of resources only if those inequalities benefit the worst off. There is an important point here to clarify: while those without disabilities tend to view individuals with disabilities as ‘worse off’ and assume the association of disabling traits with a lower quality of life, disability advocates would argue otherwise, stressing that disabled people experience disadvantage precisely because of this type of biased view. While biomedical references are useful in assessing quality of life, inequities may arise from the very application of these ableist paradigms to those with disabilities. Social models of disability variously argue that *social contexts* create disadvantages and that physical differences are not the sole root of attitudinal barriers [24]. The WHO definition of disability states that “disability is a part of being human”, and in doing so captures the inter-relationship between a different body or health state and personal or environmental factors such as inaccessible infrastructure and/or social stigma [25]. A given impairment is thus not a stable entity, and will manifest differently depending on the individual and their contextual environment. This includes the environment of the clinic, and the ways in which an individual’s differences interact with the health system structures and the cultures of clinicians. Disability philosopher Joel Michael Reynolds argues:

Given that the vast majority of clinicians are able bodied, this means that many, if not most, clinicians mischaracterize the quality of life of people with disabilities[…]. People with disabilities, on the whole, flourish in all sorts of bodies and in all sorts of ways.

What many people with disabilities do report as diminishing quality of life is often less the direct effect of their physical or psychological impairments than the effects of living in a society that is designed for and supportive of able-bodied people alone [20]. Using an approach of equality of opportunity, healthcare can be argued to have a *positive* obligation to correct for undeserved disadvantages imposed by society on individuals with disabilities, even if doing so creates inequalities in resource allocation. Presently, disabled people (like other marginalized groups) attain lower rates of education, employment, housing, and income than their able-bodied counterparts [27, 28]. As they currently stand, many triage protocols reiterate and reify this disparity. Just as we use quotas and forms of affirmative action to counteract prejudicial practices toward racialized and gendered persons, fair equality of opportunity demands that we apportion medical care in a manner that is devoid of discrimination and *ableism*. Just as we cannot ethically countenance removing health care resources from racialized minorities because they have suffered higher rates of morbidity and mortality than other populations with COVID-19 [27], we ought not do so for disabled people on the basis of assumptions that they will fare poorly when infected.

### Capabilities theory

Capabilities theory recognizes that justice requires the fulfillment of central human capabilities which are conducive to a flourishing life lived with dignity. These capabilities include: life, bodily health, bodily integrity, senses, imagination, and thought; emotions; practical reason; affiliation; interactions with other species; play; and control over one’s environment [29]. Martha Nussbaum, a pioneer of the theory, argues that all capabilities are requisite to a life worthy of human dignity, and that it is the duty of society to provision the means necessary for its participants to satisfy these capabilities.

Applying the capabilities approach to the realm of disability, it is apparent that physical- or neuro-diversity does not diminish most of this list but rather highlights the importance of considering each feature in an inclusive manner. Studies demonstrate the extreme adaptivity of our species: for example, even individuals who experience quadriplegia as the result of spinal cord injury report experiencing a similar level of happiness as prior to their injury (it should be noted, with a lived experience which may be vastly different than perceived by able-bodied persons, even by the individual involved prior to their injury) [30]. A congenitally disabled person may have an even firmer sense of bodily integrity and personal satisfaction, given their own existence is all that they have experience of. Individuals living with disability variously experience the planet, their emotions, reason, attachments, play, and ambition—the human experience—in a manner that is of equivalent importance to the experiences of others around them.

Grounding bioethical triage principles in the focus of traditional liberal theory on reason and independence means that we rely on an impoverished vision of the human being. By employing capabilities theory in triage, we adopt a richer conception of ourselves that enables fulsome understanding of how we might allocate resources in a manner in keeping with fairness across our whole human community. Applied to the pandemic context, the capabilities approach articulates a conception of justice requiring not only the absence of negative hindrances that obstruct an individual’s ability to satisfy these capabilities, but also the provision of positive freedoms in support of flourishing [15]. In many ways, the focus of the capabilities approach is similar to that of an international human rights framework—in fact, this framework has been adopted by many international organizations [29, 31, 32]. Nussbaum writes:

In some form, [all ten capabilities] are held to be part of a minimum account of social justice: a society that does not guarantee these to all citizens, at some appropriate threshold level, falls short of being a fully just society, whatever its level of opulence [29].

The capabilities approach and disability rights critique of clinical triage protocols highlight the need for a richer conceptualization of our frameworks for selfhood, justice, and freedom. The purpose of adopting a capabilities approach is not to create a “checklist” of qualities required to be human, but rather to challenge the traditional Enlightenment dogma of justice, and expand our focus beyond the negative freedoms (rights) often inscribed in procedural frameworks, toward positive freedoms conducive to the achievement of meaningful life goals and experience. Though seemingly idealistic, the latter calls into question notions of human rights and public duties to provide a more nuanced idea of what justice entails.

### Procedural justice

In addition to adopting richer notions of justice, we must also consider the issue of fairness in the process of protocol development itself. Individuals with disabilities (particularly severe mental, physical, and cognitive impairments) have historically been excluded from participation in the protocol development process, and in choosing the principles that inform them [33]. We argue that the exclusion of people with disabilities from policy discussions surrounding resource allocation has contributed to the inadequate consideration of their interests. The first wave of the current pandemic disproportionately impacted people with pre- existing morbidities, chronic illness, and disabilities [34–36], despite more cautious behaviour on the part of these individuals due to expected vulnerability to illness [37]. In North America, a majority of deaths have occurred in long-term care facilities [38]. Given that physical and mental dysfunction increase with age, and both the elderly and those with disabilities more often live in congregate settings, the disproportionate rates of infection, morbidity, and mortality in these groups did not result solely from increased medical frailty, but as much from policies which encouraged the seeding of the virus in these vulnerable communities via decisions limiting personal protective equipment and admissions to acute care [39–41].

Given mounting criticism, several groups have recommended that protocols be developed and revised in collaboration with individuals with disabilities and their representatives, ensuring an ethical and just procedure through which guidelines are created and distributed [4–6]. Some have proposed a need to consult with human rights experts and marginalized communities who have been disproportionately impacted by COVID-19 [5], also highlighting the need for greater transparency and openness in the distribution of these clinical guidelines.

The importance of procedural ethics in pandemic planning had been articulated previously. In the aftermath of the SARS-CoV-1 outbreak, stakeholder engagement and timely public debate were identified as necessary for any ethical framework to be “relevant and legitimate” [42]. Despite this historical lesson, triage protocols continue to be developed in relative secrecy. The draft Ontario protocol, for example, only entered into public knowledge following its coverage in a news article, despite having already become active to quietly inform clinical practice [43]. While we recognize that public circulation makes these protocols vulnerable to scrutiny, procedural justice requires that triage decisions are made transparently, with input from the diverse members of society who will be affected by them.

The demands of procedural justice may require that triage decisions be further removed from the bias of the clinical context and vested instead in robust and equitable guidelines. The mantra of the disability movement, “nothing about us without us,” arose from beliefs and practices in the medical community that presumed that disabled persons requiring care provision could not autonomously direct their own lives—as if the propensity to self-determination should be based solely on one’s ability to perform activities of daily living. Indeed, social and health institutions play crucial roles in the lives of persons with disabilities, and because the vast majority of clinicians are able-bodied, it is absolutely critical that disabled people be included in the discussion about what is a fair allocation of limited clinical resources. Rather than holding their interests and needs at arm’s length, fairness of opportunity requires that disabled persons be expressly present and heard at the table. This can be a potential remedy to their ongoing disadvantage, bringing greater equity to the triage process and protecting physicians from bearing the burden of agency at the point of care [33].

## Recommendations for justice in the pandemic and beyond

Ensuring a just pandemic response is but one aspect of ensuring a just society more broadly. Medical practice can increasingly return individuals to the community with ‘manageable’ conditions that might have been fatal a generation ago. Defining who is ‘disabled’ is a fluid and inexact exercise and is difficult to capture under one homogenous reference; however, it is clear that disability in its many guises is an immutable feature of modern life. Clinicians have an ethical responsibility to attend to the dignity of such people who comprise a growing proportion of their practice and society as a whole.

### Guidelines for pandemic clinical triage protocols

Here, we propose that efforts to guide justice in medical decision-making require a more granular conception of justice that encompasses both substantive and procedural considerations, as well as the inclusion of positive freedoms as founding duties of both medicine and society. While a strictly medical perspective may require a physician to consider the frailty of a patient in terms of the amount of independence they maintain or the care they receive—often with respect to “activities of daily living”—a social model of disability and a capabilities approach demands that the clinician’s assessment be less fixated on individual autonomy and more on individual capabilities and flourishing. To provide a concrete example, the renowned physicist Stephen Hawking would have scored very heavily on such a frailty scale, while also scoring very highly on an assessment of capability and flourishing—we argue the latter would be more appropriate. This means understanding that human dignity is not necessarily defined by one’s level of independence from others, but rather by a person’s connection to community, nature, and their overall sense of well-being, and prioritizing the latter in our clinical approach.

Our review has identified a number of limitations in the Canadian COVID-19 triage documents with respect to our proposed approach. Recognizing that there may be resistance to the perspectives endorsed in this article, we hereby suggest a number of broad recommendations that can guide the revision and creation of increasingly justifiable triage protocols (Table 1).

**Table 1:** Ethical recommendations for clinical triage and guidelines

Recommendation	Implications
1. Frameworks of justice and utility underlying the COVID-19 triage protocols must be explicitly defined and explored	Vague and nonspecific terms such as “utility” and “fairness” must be clearly defined and operationalized into concrete metrics (e.g., utility through maximizing life-years saved, fairness through prioritizing the worst off)
2. Principles of transparency, openness, and procedural justice must be followed in developing clinical decision- making documents. An explicit commitment to diversity and inclusion must be incorporated into procedural justice considerations	The development of protocols should be done in the public sphere, with public stakeholders and input from members of disability communities to ensure procedural justice. Recommendations should be made with guidance from disabled individuals and their advocates, including caretakers and disability rights groups
3. Triage policies must adhere to human rights standards and explicitly condemn bias and discrimination	Ableist biases which categorically exclude individuals with certain disabilities (e.g., advanced neurocognitive disorder) must be prohibited. Clinical biases surrounding quality-of-life assessments which negatively affect individuals with disabilities must be recognized and explicitly condemned
4. Reasonable accommodations must be made in consideration of disability status, and how an individual’s underlying conditions may influence their COVID-19 care needs	Policies should be written and revised to provide reasonable accommodations for individuals with disabilities. These may include allocating greater time on ventilator support for equal chances of survival, or providing communication assistance for those who are Deaf or Deaf-blind
5. Clinical scores and prognostic instruments must be carefully examined to exclude factors such as functional status that may not accurately represent prognosis in people with disabilities	Clinical criteria that are value-neutral at face value but disproportionately affect individuals with disabilities (e.g., evaluation of long-term survival, triage based on co-morbidities) should be reconsidered. Proper adjustments should be considered in the application of these clinical criteria to disabled individuals
6. COVID-19 care and triage must not be seen in isolation but must be seen within the broader context of justice for people with disabilities	Protocols surrounding scarce resource allocation carry a powerful symbolic meaning: one which embodies the value judgements that are made by society in critical times. Since these policies touch on fundamental human rights—such as the right to life, to health care, and equality—they must be drafted and treated as such

We anticipate a counterargument to our recommendations to come from the understanding that clinicians currently employ clinical criteria to deny or withdraw life-saving care (e.g., in cases of futility) to allocate scarce resources. With regards to clinical decision-making, we argue that there is a morally significant difference between the provision of care on a case-by-case basis and the integration of pandemic triage policies affecting individuals on a population-level. While the former requires a clinician to act in the best interests of an individual patient, the latter requires consideration of duties towards the patient herself in addition to responsibilities towards society at large (e.g., acting as a steward of the broader system). When policies seemingly predicated on “objective” clinical criteria systematically disadvantage certain groups of individuals on a macroscopic level, it creates grounds for inequitable treatment and injustice at large.

#### Justice beyond pandemic triage

While triage and the allocation of scarce resources always represent a tension between social utility and individual rights, the question of what traits ought to be considered as “justified” limits to one’s access to key resources is vital. Decisions which seek to maximize clinical outcomes are often laden with value judgements which extend beyond consideration of clinical criteria alone and may factor in ableist prejudices. This is observed in the field of organ transplantation, a practice which has historically focused on allocating scarce resources to maximize utility. Algorithms used to select candidates for organ transplantation often rely on one’s short and long-term probability of survival with the life-saving intervention. Although several American states have passed laws banning transplant discrimination on the basis of disability, 85% of pediatric transplant centers nonetheless consider neurodevelopmental disability when determining transplant eligibility, despite little data to suggest that such disabilities result in worse outcomes [44, 45]. In 2019, the National Council on Disability issued a statement regarding transplant discrimination which bears striking similarity to the open letters drafted in light of the current pandemic, stating: “at the heart of the debate are concerns about scarcity of transplantable organs and societal beliefs about the worth of the life of a person with a disability” [46]. Both clinical triage protocols and transplant allocation algorithms reveal that criteria deemed to focus on probability of survival nonetheless carry significant bias against individuals with disabilities, even when such disabilities may not affect clinical outcome.

It is often impossible to separate clinical criteria from social and demographic ones. While triage guidelines such as the Ontario draft protocol often include statements that decisions should be “based on clinical considerations, integrating all relevant information, and not solely on any demographic or socioeconomic factor” [1], social determinants of health often predispose some individuals to clinical need, and their clinical need is subsequently weighed “in isolation” (against that of other patients) to determine priority and, often, deprivation of care. Clinical decision-making must not be considered in isolation of the broader landscape of health, ability, and care, and the requirement to actively engage with these factors is a crucial component which applies to triage and beyond. In recognizing that clinical outcomes and social determinants of health are intertwined, to promote a more equitable framework for justice requires us to not only re-evaluate the bias found in clinical assessment tools such as the CFS and SOFA score, but also to promote positive freedoms afforded for individuals with poorer prognoses attributed to social conditions (i.e., poverty, race, disability). We propose that this ought to occur in the form of reasonable accommodations (Table 1). A comprehensive exploration of what reasonable accommodations entail is beyond the scope of this paper, but we believe that it should be outlined with strong input from the communities so closely affected by them.

It should be noted that the impetus for our critique was initially borne out of the inequities found in many Canadian clinical triage guidelines following the first wave of SARS-CoV-2 in 2020. Since then, many jurisdictions have changed their rationing criteria in response to activists, including the Office for Civil Rights at HHS in the United States which has rejected the explicit discrimination of individuals with disabilities in their crisis care standards. However, several jurisdictions continue to ration on the basis of age and disability in more implicit ways. Our intention is not to provide a review of clinical triage protocols at large, but rather to highlight the ways in which these protocols perpetuate ableist norms in clinical practice, and to propose a more equitable framework for justice.

## Conclusion

The disability critique highlights a need to enrich the conceptions of justice and freedom that underpin modern triage protocols. Disability rights activists have long objected to narrow conceptions of human rights that fail to account for the richness of seemingly ‘marginal’ lives. To borrow from the renowned civil rights lawyer, Harriet McBryde Johnson:

The widespread assumption that disability means suffering feeds a fear of difference […] We need to confront the life-killing stereotype that says we’re all about suffering. We need to bear witness to our pleasures. [42]

Sterile and non-specifically phrased guiding ethical principles are an inadequate means of informing clinical decision-making in critical scenarios, and current formulations invite the criticism that many such existing protocols serve to disadvantage individuals with disabilities. Many of these approaches have taken a narrow definition of justice, heavily laden with bias. A more explicit model of justice informed by positive freedoms is the first step in repairing current protocols which fail to guide just and equitable decision-making.

## Supplementary Information

**Additional file 1: **Overview of clinical guidance documents and disability rights objections.

## Data Availability

All data generated or analyzed during this study are included in this published article.

## Abbreviations

CFS: Clinical frailty score
SOFA: Sequential organ failure assessment
ADL: Activities of daily living
COVID-19: COVID-19/ SARS-CoV-2
WHO: World Health Organization
