# Supplementary material for: An ethical analysis of clinical triage protocols and decision-making frameworks: what do the principles of justice, freedom, and a disability rights approach demand of us?
Source: BMC Med Ethics. 2022 Feb 11;23:11. doi: 10.1186/s12910-022-00749-0 (PMC8831871; doi:10.1186/s12910-022-00749-0)
Supplement: Supplementary file 1 — Additional file 1: Overview of clinical guidance documents and disability rights objections. [file 12910_2022_749_MOESM1_ESM.docx]

**Additional Table 1. Overview of clinical guidance documents and disability rights objections**

| **Geographic region** | **Document** | **Summary** | **Complainants and objections** |
| --- | --- | --- | --- |
| Ontario | **Clinical Triage Protocol for Major Surge in COVID Pandemic**  Authors: Ontario Health  <https://caep.ca/wp-content/uploads/2020/04/Clinical-Triage-Protocol-for-Major-Surge-in-COVID-Pandemic-March-28-202.pdf> | **Guiding Principles:**   1. **Utility:** to derive maximum benefit from allocating resources to those who derive the greatest incidental benefit 2. **Proportionality**: number of individuals negatively affected by triage system should not exceed what would be required to accommodate the surge in demand 3. **Fairness:** clinically-relevant criteria should be used to allocate resources   Clinical triage criteria  **Exclusion criteria:**   - Low probability of surviving acute illness - Low probability of surviving more than a few months regardless of acute illness   These are deemed to reflect utility and fairness  Among these are people with:   - Severe baseline cognitive impairment (unable to perform activities of daily living due to cognitive impairment) - Advanced irreversible neurodegenerative disease | Open Letter: Ontario’s COVID-19 Triage Protocol (<https://archdisabilitylaw.ca/wp-content/uploads/2020/04/April-8-2020-Open-Letter-Ontarios-COVID-19-Triage-Protocol-PDF.pdf>)  Recommendations:   1. People with disabilities cannot get deprioritized for critical care on basis of disability (e.g. cognitive disabilities, advanced neurodegenerative diseases, etc) 2. Triage Protocol must clearly state that clinical judgement must not be informed by bias, stereotypes, or ableism 3. Persons with disabilities cannot be deprioritized for critical care based on the supports they receive for daily living 4. Triage protocol must ensure that persons with disabilities receive necessary disability-related accommodations |
| Quebec | **Triage for Access to Intensive Care and Allocation of Resources Such as Ventilators in a Pandemic**  Authors: Marie-Eve Bouthillier, PhD for the Assistant Deputy Minister of the Quebec Health and Social Services  <http://www.cmq.org/pdf/coronavirus/msss-protocole-national-triage-soins-intensifs-pandemie-def.pdf> | **Guiding Principles:**   1. **Maximization of benefits:** deriving maximum benefits for a community by prioritizing resources for those who derive the greatest additional benefit 2. **Proportionality:** number of people negatively affected should not exceed what would be required to accommodate the surge in demand, and should not be more negative than the number of people who would be affected using a “first come first serve” approach 3. **Transparency and trust:** widely available, clearly documented in terms of accountability, consistently applied 4. **Efficiency and durability:** sorting criteria has to be simple enough to use at bedside 5. **Equity:** prioritization should not be granted on basis of socioeconomic privilege or political rank   Clinically relevant criteria should be first used  **Exclusion criteria:**   - Criteria that indicates low probability of surviving acute illness - Criteria that indicates low probability of surviving more than a few months   **Step 1:** exclusion of any patient whose estimated mortality is greater than 80% **Step 2:** exclusion of any patient whose estimated morality is greater than 50%  **Step 3:** exclusion of any patient whose estimated mortality rate is greater than 30%  Exclusion criteria include: severe cognitive impairment, advanced and irreversible neuromuscular disease (e.g. Parkinson’s, ALS), decreased functional capacity according to clinical frailty score (Geriatrics)   - Functional limitations and severe cognitive impairment may have an impact on individuals with intellectual disabilities | Open Letter: Triage Quebec Initiative  <https://triage.quebec/en/the-icu-triage-protocole-in-quebec/our-recommandations/>  Recommendations:   1. That the exclusion criteria referring to specific handicaps (such as cognitive impairment, Parkinson’s disease and amyotrophic lateral sclerosis) be removed from the Protocol; 2. That clinical evaluation tools relating to functional autonomy (such as the Clinical Frailty Score or the Functional Autonomy Measuring System) not be used to exclude a patient from intensive care; 3. That the Protocol offers the guarantees necessary for preventing discriminatory prejudices on the value of a disabled person’s life being included implicitly during the triage process. An explicit statement to that effect should also be included; 4. That the necessary accommodations for the disability will be in place, during and after critical care, so that people with disabilities have an equal opportunity to receive, understand and benefit from this care; 5. That persons with disabilities and their representatives can participate in the revision of the Protocol; 6. That the Ministry of Health and Social Services be more transparent about the triage process for access to intensive care implemented in Quebec, in order to preserve public confidence. |
| British Columbia | **COVID-19 Ethical Decision-Making Framework**  Authors: BC Ministry of Health  Provincial COVID-19 Task Force  <https://www2.gov.bc.ca/assets/gov/health/about-bc-s-health-care-system/office-of-the-provincial-health-officer/covid-19/ethics_framework_for_covid_march_28_2020.pdf> | **Ethical principles and values:**   - **Respect:** individual autonomy, individual liberties, cultural safety   - Respect for privacy and confidentiality   - Obligation on behalf of leaders and care providers to be truthful and honest - **Harm principle:** society has right to protect itself from harm - **Fairness:** persons ought to have equal access to health care resources, however   - **Equity**: those who most need and can derive greatest benefit ought to be offered resources preferentially   - **Utility & efficiency:** resources ought to be distributed such that the maximum benefits to greatest number is achieved   - **Consistency:** same application across populations regardless of individual factors (e.g. race, age, disability, ethnicity, ability to pay, SES, etc) - Least coercive and restrictive means - Cooperation - Reciprocity - Proportionality - Flexibility - Procedural justice:   - Openness and transparency   - Inclusiveness   - Accountability   - Reasonableness | Letter from Disability Alliance BC  <https://disabilityalliancebc.org/wp-content/uploads/2020/04/Letter-to-Minister-Dix-Re-Triage-Protocol.pdf>  Recommendations:   1. In developing the protocol, the government must consult with human rights experts and marginalized communities who have been disproportionately impacted by the crisis 2. People with disabilities must not be deprioritized for critical care based on their disabilities or on the supports they receive for daily living    1. Need to use only objective clinical criteria directly associated with COVID and not stereotypes or assumptions about a person’s disability or longer-term mortality rates    2. Some people with disabilities need accommodation and assistance 3. Protocol must clearly state that clinical judgement must not be informed by bias, stereotypes, ableism. Must ensure that people with disabilities have equal opportunity to receive, understand, and benefit from critical care |
| Canada-wide | **COVID-19 pandemic guidance for the health care sector**  Authors: Government of Canada  <https://www.canada.ca/en/public-health/services/diseases/2019-novel-coronavirus-infection/health-professionals/covid-19-pandemic-guidance-health-care-sector.html#a45> | Ethical considerations:   - Trust and solidarity - Reciprocity - Stewardship - **Equity and fairness**   - Benefits and risks should be fairly distributed   - “When health care resources are in extremely short supply, fair distribution may entail a strategy where individuals most likely to survive will be prioritized to **maximize the benefits**” - **Good decision-making process:** open, transparent, accountable, inclusive   Section 4.5 Allocation of Scarce Resources  Considerations:   - All hospitals in region or province should implement uniform resource allocation process - **Prioritization guidelines should be evidence-based and objective** - There should be no difference in allocating scarce resources between patients with COVID and those with other medical conditions - Experienced physicians should function as triage officers but not be involved in triage and in direct care of patients requiring triage - Appeals should be limited to concerns about procedural mistakes - Patients should be provided dignity and comfort | Open Letter: COVID-19 Triage Protocols & the Rights of People with Disabilities  <https://www.include-me.ca/covid-19/resource/open-letter-covid-19-triage-protocols-rights-people-disabilities>   - People with disabilities and Deaf people are at special risk: some are vulnerable to COVID-19 and all are vulnerable to discriminatory triaging - We urgently need rights-based, non-discriminatory guidelines for provision of health care in this pandemic - Guidance to health care providers must respect the human rights of all people, including persons with disabilities   Recommendations:   1. Assumptions about current or future quality of life of a person with a disability is not a valid reason to deny access to health care for COVID-19 2. Existing disability-related conditions unrelated to the chance to benefit from treatment must not play part in decision-making 3. Need for and use of disability-related supports and anticipated future costs of these supports must not play any role in assessing access to tx 4. Person’s need for support to make healthcare decisions must not be used as a reason to limit or deny access to tx 5. Guidelines for assessing, providing, and evaluating health care must be developed with organizations of people with disabilities and their families. 6. The assessment, provision, and evaluation of health care must be conducted with full accessibility and equality of communication, including the provision of qualified professional Sign language interpreters for people who are Deaf and Deaf-blind. |
| Canada-wide | **Framework for Ethical Decision Making During the Coronavirus Pandemic** | Goal: to provide Canadian physicians with guidance in case they find themselves in positions of having to triage between seriously ill patients  Process: use of documents, reports, and policies produced by other individuals and countries  Recommendations:   1. Maximize benefit: priority for resources should save (1) more lives and (2) save more life years  - Reference to utilitarian ethical principles which emphasize population outcomes and non-utilitarian views that emphasize of each human life - Maximize number of patients that survive tx with reasonable life expectancy as primary aim  1. Critical interventions should go to front-line workers first 2. If patients have similar prognoses, equality should be invoked and operationalized through random allocation 3. Prioritization guidelines should differ by intervention and respond to changing scientific evidence  - Maximizing benefits may justify consideration of prognosis (how long patient is likely to live if treated) which may lead to prioritizing younger patients with fewer comorbidities  1. Those who participate in research should receive priority for Covid-19 interventions 2. There should be no difference between allocating scarce resources to patients with Covid-19 and other medical conditions |  |
